# 

**Published:** 1857-11

**Authors:** 


### SUBSCRIPTIONS RECEIVED.

J. H. Ethridge, Ga., 3d vol.; R. M. Williams, Ga., 3d vol.; Jno. A. Moody, Ga., 3d vol.; G. W. Powell, Ga., 3d vol.; A. A. Davis, Va., 1st and 2d vols.; A. B. Wallace, Ga., 3d vol.; R. J. Bigelow, Fla., 3d vol.; David Kendall, Ga., 2d and 3d vols.; G. W. Burney, Ga., 2d vol.; W. A. Speir, Ga., 3d vol.; W. T. Sanders, Ga., 3d vol.; W. S. Swanson, Ga., 3d vol.; W. P. Anderson, Ga., 3d vol.; G. L. Johnson, Ga., 3d vol.; James T. Bond, Ga., 3d vol.; A. M. Parker, Ga., 3d vol.

### TO CORRESPONDENTS.

☞ All Communications pertaining to the Editorial Department of the Journal, hould be addressed to the Editors.

☞ All Communications, having reference to the Subscription, the Advertising or the Financial Department, should be addressed to G. P. EDDY & Co

